# Evaluation of Different Strategies to Improve the Management of *Helicobacter pylori* Infection at the Primary Care Level: Training Sessions Increase Prescription Appropriateness of Treatment Regimens

**DOI:** 10.3390/antibiotics11121746

**Published:** 2022-12-03

**Authors:** Enrique Alfaro, Samuel J. Martínez-Domínguez, Viviana Laredo, Ángel Lanas, Carlos Sostres

**Affiliations:** 1 Department of Gastroenterology, Lozano Blesa University Hospital, 50009 Zaragoza, Spain; 2 Aragón Health Research Institute (IIS Aragón), 50009 Zaragoza, Spain; 3 School of Medicine, University of Zaragoza, 50009 Zaragoza, Spain; 4 CIBER for Liver and Digestive Diseases (CIBERehd), 28029 Madrid, Spain

**Keywords:** *Helicobacter pylori*, training sessions, specific counselling, primary care level

## Abstract

*Helicobacter pylori* infection (*H. pylori*) is mainly managed at the primary care level. Our group previously performed a study demonstrating that providing specific counselling (SC) to primary care practitioners (PCPs) who requested a urea breath test (UBT) improved treatment management but not indications for *H. pylori* tests. SC was given in the form of a personal letter addressed to PCPs with UBT results which contained information about accepted UBT indications and a *Helicobacter pylori* treatment algorithm. The purpose of the present study was to evaluate the effect of training sessions (TS) on UBT indications, antibiotic prescriptions and eradication rates. This was a quasi-experimental study performed at primary care centres (PCCs). Phase I included 399 patients diagnosed with *H. pylori* infection after providing SC to PCPs. Phase II included 400 *H. pylori*-positive patients after giving TS to PCPs who had already received SC (100 from PCCs with TS and 300 from PCCs without TS). An improved trend in the appropriate indication of *H. pylori* diagnosis was observed between Phase I and PCCs with TS in Phase II (57.5% vs. 67%; *p* = 0.06). TS improved appropriate prescriptions in PCCs with TS compared to PCCs that only received SC in Phase I and II (94% vs. 75.3%, *p* = 0.01; 94% vs. 85.6%, *p* = 0.04, respectively). Eradication rates showed no differences between groups. In conclusion, training sessions after specific counselling improved antibiotic prescription appropriateness but not eradication rates.

## 1. Introduction

*Helicobacter pylori* (*H. pylori*) is a Gram-negative bacterium that colonises the gastric epithelium in approximately half of the world’s population. Despite its ubiquitous distribution, the prevalence among different geographical areas is heterogeneous [1,2,3,4]. *H. pylori* infection has been associated with chronic gastritis, peptic ulcers, gastric adenocarcinoma and mucosa-associated lymphoid tissue (MALT) lymphoma, so successful eradication can prevent the development and progression of these diseases [5]. 

Although most patients remain asymptomatic, *H. pylori* can cause dyspepsia, which is one of the most frequent reasons for consultation related to the digestive system, which prompts *H. pylori* investigation in a large number of patients in daily clinical practice [6,7]. Therefore, an adequate diagnostic–therapeutic strategy is essential to avoid over-diagnosis, unnecessary consumption of resources and increased antibiotic resistance. For this purpose, different consensus conferences have published recommendations at the national or international level (IV Consensus Conference on *H. pylori* infection treatment and Maastricht V Conference, respectively) [8,9].

Data from the European Registry on the Management of *H. pylori* (Hp-EuReg) showed *H. pylori* resistance to clarithromycin above 15% in naïve patients between 2013 and 2020. In addition, resistance to levofloxacin and dual and triple therapy resistance were also high [10]. In this context, it is a challenge to achieve the effectiveness threshold of 90% or above required for antibiotic regimens. In fact, daily clinical practice studies performed in recent years found effectiveness rates far from this target [11].

In recent years, *H. pylori* infection management has been progressively transferred to the primary care level. McNicholl et al. and Cano-Contreras et al. reported suboptimal results at the primary care level in the past few years, suggesting the need for new strategies to optimise adherence to recommendations [12,13]. 

In this context, different educational strategies focused on the patient have been evaluated, finding contradictory results. A retrospective study assessing patient–doctor interaction by a mobile messaging application in China failed to demonstrate an improvement of eradication rates, compliance and incidence of adverse effects [14]. However, a prospective randomised controlled trial evaluating the effect of twice-daily short message-based re-education showed an improved eradication rate in young Chinese people [15]. A meta-analysis found that patient education strategies have positive effects on both *H. pylori* adherence and eradication rates [16]. 

Nevertheless, few studies have been specifically developed at the primary care level. Therefore, our group previously carried out a study at the primary care level demonstrating that sending written specific counselling (SC) to primary care practitioners (PCPs) who requested a urea breath test (UBT) significantly improved treatment management, but not the indications for *H. pylori* tests. Consequently, it seems necessary to develop new strategies to optimise the diagnosis and management of *H. pylori* infection [17].

The aim of this study was to assess the effect of giving face-to-face training sessions (TS) to PCPs, in different stages of *H. pylori* management, after sending them written specific counselling (SC).

## 2. Results

### 2.1. Baseline Characteristics

A total of 799 patients with positive UBT requested from primary care centres (PCCs) were included in the analysis: 399 consecutive cases pertained to Phase I, where PCPs received written SC, and 400 consecutive cases were recruited after the TS period (Phase II), starting one month after giving the TS. In Phase II, 100 UBT requests came from PCCs that previously received TS and 300 UBTs from PCCs without TS. The mean age of participants was 47.33 ± 15.68 years, and 517 (64.7%) were women.

### 2.2. Indications

Dyspepsia without alarm signs in patients younger than 55 years and confirmation of eradication represented the vast majority of appropriate UBT indications. Gastroesophageal reflux-related symptoms, dyspepsia with alarm symptoms or in patients 55 years or older, and abdominal pain without dyspepsia features were the most frequent inadequate indications in both phases. Phase I showed numerically lower rates of appropriate indications compared to PCCs with TS and PCCs without TS (57.5% vs. 67%, *p* = 0.060; 57.5% vs. 62.7%, *p* = 0.314, respectively). No significant differences were found when PCCs with and without TS were compared (67% vs. 62.7%, *p* = 0.457) (Table 1).

### 2.3. Prescription Appropriateness

In Phase I, 76.2% (304/399) patients with a positive UBT received antibiotic treatment. In Phase II, patients who received antibiotic treatment increased up to 83.5% globally (334/400) (*p* = 0.01). No differences were observed between PCCs with TS and without TS in Phase II (84% [84/100] vs. 83.3% [250/300], *p* = 0.87). 

**Figure 1:**
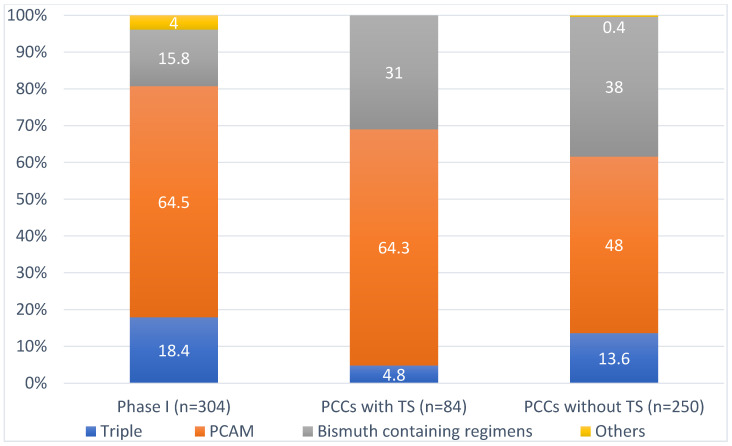
Antibiotic regimen prescribed. TS: training sessions, PCCs: primary care centres.

PCAM (proton pump inhibitor (PPI), clarithromycin, amoxicillin and metronidazole) was the most widely used regimen in Phase I (64.5%) and Phase II in PCCs with and without TS (64.3% and 48.0%, respectively). Use of bismuth-containing regimens (single capsule bismuth containing or PBMT: PPI, bismuth salts, metronidazole and tetracycline) increased in Phase II (15.8% vs. 31.0% in PCCs with TS and 38.0% in PCCs without TS). On the other hand, triple therapies, which are not indicated in current national consensus documents, were reduced from 18.4% in Phase I to 4.8% in PCCs with TS and to 13.6% in PCCs without TS (Figure 1). 

Improvement of prescription appropriateness was observed in Phase II when compared to Phase I (Phase I vs. PCCs with TS: 75.3% [229/304] vs. 94% [79/84], *p* = 0.012; Phase I vs. PCCs without TS: 75.3% vs. 85.6% [214/250], *p* = 0.0001). Indeed, higher rates of prescription appropriateness were found comparing PCCs with TS vs. PCCs without TS in Phase II (94.0% vs. 85.6%, *p* = 0.04). 

### 2.4. Eradication Rates

The absence of confirmatory UBT in patients who received antibiotic treatment (which is mandatory to confirm *H. pylori* eradication) was a frequent finding in all groups: Phase I—29.3% (89/304); PCCs with TS—31.0% (26/84); PCCs without TS—25.6% (64/250). TS did not improve the performance of eradication control UBT (69% in PCCs with TS vs. 74.4% in PCCs without TS, *p* = 0.33).

**Figure 2:**
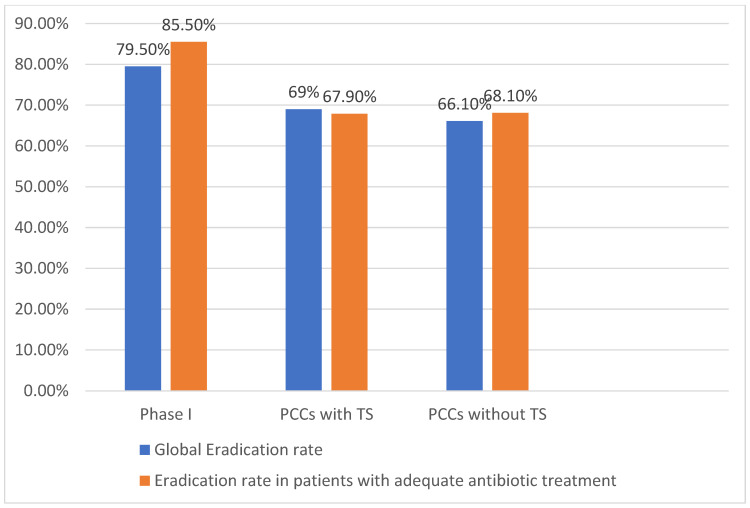
Eradication rates. PCCs: primary care centres; TS: training sessions.

Overall, no improvement in eradication rates was observed when comparing Phase I with Phase II PCCs with TS (79.5% [171/215] vs. 69% [40/58], *p* = 0.06) or between Phase I and PCCs without TS (79.5% vs. 66.1% [123/186], *p* = 0. 14). No differences in eradication rates were observed between groups in Phase II (69% vs. 66.1%, *p* = 0.68) (Figure 2).

Eradication rates were influenced by prescription appropriateness. In Phase I, the eradication rate was significantly higher in patients with an adequate antibiotic treatment (85.5% [142/166] vs. 58.3% [28/48], *p* = 0.001). In Phase II, no differences were observed in eradication rates according to antibiotic appropriateness. 

In Phase II, patients with an adequate antibiotic treatment from both groups had eradication rates significantly lower compared to Phase I (85.5% vs. 67.9% [38/56], *p* = 0.01 in PCCs with TS; 85.5% vs. 68.1% [109/160], *p* = 0.002 in PCCs without TS).

No differences were observed in eradication rates between PCCs with TS and without TS (67.9% vs. 68.1%, *p* = 0.97) (Figure 2).

## 3. Discussion

Today, in many countries, *H. pylori* infection is mainly managed by PCPs instead of gastroenterologists [17,18]. It is important to know the management of *H. pylori* infection by PCPs, as it allows us to develop strategies to improve the diagnosis and treatment of the infection based on current national and international guidelines and consensus documents [8,9,19,20]. Proper management has a significant impact on healthcare systems, since overdiagnosis and inadequate antibiotic treatment can lead to an increase in global antibiotic resistance.

Previously, some studies reported that face-to-face training sessions were an effective tool to improve the clinical practice of healthcare professionals, and the use of printed educational material can also help to achieve this goal [21,22]. In this way, some strategies have been successfully developed to improve *H. pylori* management at the primary care level [23,24]. However, most of these strategies mainly focus on the appropriate use of antibiotic treatments and do not evaluate adherence to diagnostic indications accepted by guidelines or, more importantly, if these strategies improve eradication rates [23].

In this study, the appropriateness of UBT indications was suboptimal at the primary care level. We found a high rate of inadequate UBT requests in both phases. The most common inappropriate UBT indications were gastroesophageal reflux-related symptoms and dyspepsia with alarm symptoms or in patients older than 55 years, similar to other studies reported in different countries [13,25,26]. Current guidelines do not support testing or treating *H. pylori* infection in patients with gastroesophageal reflux symptoms. Moreover, dyspepsia in patients older than 55 years involves performing an upper gastrointestinal endoscopy as the first medical exam because the risk of gastric cancer increases from that age, according to national guidelines [9,19]. 

A previous study carried out by our group reported that providing SC to PCPs who requested UBT was an ineffective strategy to improve rates of appropriate indications [17]. In the current study, we found that training sessions delivered to PCPs were not an effective strategy either, so it is necessary to develop and evaluate new strategies to improve UBT request indications, since unnecessary use of antibiotic treatments can lead to an increase in antibiotic resistance. 

Training sessions were an effective strategy to improve prescription appropriateness. The use of adequate antibiotic treatments (according to national guidelines and consensus documents) improved between Phase I and II, including in Phase I compared to PCCs without TS of Phase II (no TS in both groups). This finding could be related to the progressive acquisition over time of the knowledge included in SC by PCPs.

In this study, we detected higher rates of UBT requests for confirmation of eradication than other studies carried out in different countries. An Irish study reported that only 48% of physicians confirmed *H. pylori* eradication, data similar to those reported in Israel (43.6%) and Spain (41.8%). Studies in Pakistan (12%) and Korea (9.3%) showed even lower rates [18,25,26,27,28]. However, our rates are still suboptimal (70.7% in Phase I), and TS did not improve them (69% in PCCs with TS vs. 74.4% in PCC without TS).

Another important point is the effect of improving prescription appropriateness on eradication rates. Currently, adequate antibiotic regimens should achieve an effectiveness threshold of 90% or above; however, this objective is hardly achievable in clinical practice studies. In fact, this study found similar data to other studies previously published by our group, where an overall effectiveness of 70.7% was reported. Effectiveness varied according to the type of regimen used and according to prescription appropriateness. Triple therapies’ eradication rates were lower than 70%, whereas eradication rates with quadruple therapies were over 80% [11]. In addition, patients with adequate treatment regimens presented higher eradication rates (85.5% vs. 58.3%). 

A decrease in eradication rates in Phase II compared to Phase I (79.5% in Phase I vs. 69% in PCCs with TS or 66.1% in PCCs without TS) was observed, and although conducting clinical sessions improved the use of appropriate antibiotic regimens, this was not associated with an increase in eradication rates. This paradoxical finding could be related to a global increase in *H. pylori* antibiotic resistance. Indeed, *H. pylori* was recognised by the World Health Organisation (WHO) in 2017 as one of the 20 pathogens that can potentially become a threat to human health for this reason [29,30]. Moreover, a recent study published by Hp-EuReg containing a large number of *H. pylori* cultures demonstrated this problem, as only 44% of the patients were free of antibiotic resistance, and a high rate of resistance to commonly used antibiotics was observed in southern European countries (Spain, Italy and Greece) [10]. However, further specific microbiological studies should be conducted in our local population to confirm the relationship between low eradication rates and high rates of antibiotic resistance. On the other hand, the observed decrease in eradication rates in Phase II may also be explained by patient-related factors, such as therapeutic adherence to the prescribed regimen. 

### Strengths and Weaknesses

Despite including a prospective interventional design, the main limitations of the study are the absence of randomisation inherent to clinical trials, the small number of PCCs, the lack of antibiotic resistance data and the disparity in the sample size of both Phase II groups. However, the study has several strengths, such as a large total sample size, a prospective design and a comparison with current guidelines, which allows us to improve clinical practice based on an evidence-based quality standard.

## 4. Materials and Methods

### 4.1. Study Design

This was a quasi-experimental study assessing the effect of TS plus SC at PCCs compared to SC alone. The aims of the study were to evaluate the effect of TS plus SC on urea breath test (UBT) indications, prescription regimen appropriateness and eradication success rates at PCCs. 

The study was conducted between October 2016 and November 2019 in 57 PCCs belonging to the Lozano Blesa University Hospital National Health System area in Aragón (Spain), with a total population of 257,736 people that reflects the usual clinical practice at the primary care level. 

Inclusion criteria were age ≥18 years and current *H. pylori* infection based on a positive result of urea breath test with ^13^CO ≥ 2.5‰ (UBTest^®^, Otsuka Pharmaceutical, Tokyo, Japan). Patients were excluded in the absence of accurate information about demographic data, treatment regimen prescribed or confirmation of eradication.

**Figure 3:**
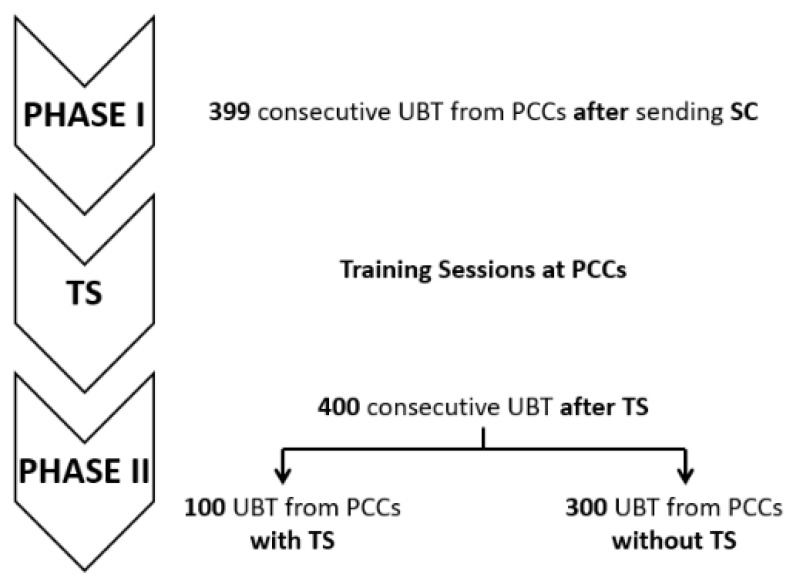
Study design and the phases that compose it. PCCs: primary care centres; TS: training sessions; SC: specific counselling; UBT: urea breath test.

The study comprised two phases (Figure 3).

#### 4.1.1. Phase I

**Figure 4:**
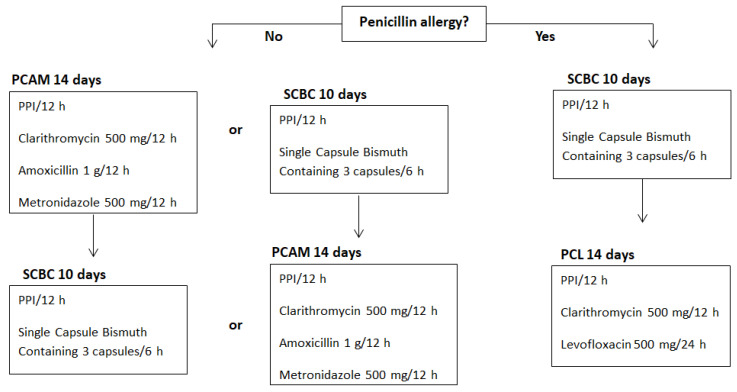
Algorithm of treatment based on the IV Spanish Consensus Conference on *Helicobacter pylori* infection treatment and consensus protocols of national scientific societies of gastroenterology and primary care physicians [8,20]. PPI: proton pump inhibitor. Modified from: Adrián Gerald McNicholl, Javier Amador Romero, Xavier Calvet Calvo, Javier Molina-Infante, Javier P. Gisbert. Diagnóstico y tratamiento de la infección por *Helicobacter pylori*. 2 Ed. Madrid: IMC; 2021 [cited 2022 Oct 10]. Available from: https://www.aegastrum-semfyc.es/require/archivos/infeccion-helicobacter-pylori.pdf (accessed on 1 November 2022).

A total of 399 consecutive UBTs requested by PCPs after sending SC were prospectively included. Our regional healthcare system provides an open-access UBT service. UBTs were requested from PCCs belonging to the healthcare area and later analysed in the Gastroenterology Laboratory of our Digestive Diseases Department. SC was a personal letter electronically addressed to PCPs and stored in the patient’s electronic medical record. It contained UBT results, accepted UBT indications (based on III Spanish Consensus Conference on *Helicobacter pylori* infection of 2016) and a *Helicobacter pylori* treatment algorithm (based on consensus protocols of national scientific societies of gastroenterology and primary care physicians) [8,20]. The national recommendations at that moment were equivalent to the Maastricht V consensus of 2016 [9]. Therefore, the appropriateness of UBT indications (Table 2) and eradication regimens (Figure 4) was based on an evidence-based quality standard.

#### 4.1.2. Phase II

After completing Phase I, one face-to-face TS was given by two gastroenterologists for approximately one hour at four PCCs with a high demand for UBTs from the same health area. TS were available for all PCPs who voluntarily wanted to participate (approximately 10 PCPs at each PCP). Additionally, supplementary printed material containing the points discussed in the TS was also delivered. The content of TS followed the current guidelines (Table 2 and Figure 4), as detailed for SC in Phase I.

After the training period, 400 consecutive UBTs were prospectively included: 100 from PCCs with TS and 300 from PCCs without TS.

### 4.2. Variables

The following variables were collected in an electronic database: age, gender, penicillin allergy, centre of origin, reason for the UBT indication, prescription date, prescribed eradication regimen and duration, agreement with current clinical practice guidelines and effectiveness. 

Effectiveness had 2 categories: success (negative eradication confirmation UBT) or failure (positive eradication confirmation UBT).

### 4.3. Statistical Analysis and Ethics Statement

First, a descriptive analysis was carried out, where qualitative variables were presented as absolute (frequency) and relative (%) values and quantitative variables were presented as mean and standard deviation (SD). Normality was assessed using the Kolmogorov–Smirnov test.

Chi square or Fisher’s exact test was performed to analyse the relationship between qualitative variables. Student’s *t*-test or the Mann–Whitney U test was used to compare means of independent groups. Efficacy analysis was performed by Modified Intention to Treat (mITT), defined to reflect the results closest to those obtained in clinical practice, which included all patients who had completed follow-up (confirmatory test available after eradication treatment), regardless of compliance. A *p* value < 0.05 was considered statistically significant. SPSS software v26.0 (SPSS Ibérica, Madrid, Spain) was used to perform data analysis.

A sample size of 384 patients was estimated, based on a confidence interval of 95%, a precision of 3% and an approximate size of the healthcare area of 250,000. Considering a loss of 5% of patients, 403 patients should be included.

All data were treated confidentially after an anonymisation process. The study was conducted according to the guidelines of the Declaration of Helsinki and was approved by the local ethics committee (code PI22/457).

## 5. Conclusions

Training sessions were an effective strategy to improve the prescription appropriateness according to current national guidelines compared to providing written SC. This improvement in prescription appropriateness did not have any impact on the global eradication rates and on patients who were prescribed adequate antibiotic treatments. In addition, training sessions did not improve the indications for UBT requests. New strategies are needed to promote knowledge, increase eradication rates and improve adherence to current guidelines at the primary care level.

## Figures and Tables

**Table 1:** Most frequent indications for urea breath test in Phase I and II.

	Phase I n = 399	Phase II n = 400	
Adequate indications, n (%)	229 (57.5)	PCCs with TS n = 100	PCCs without TS n = 300
		67 (67)	188 (62.7)
Dyspepsia	140 (61.1)	44 (65.7)	128 (68.1)
Eradication control	83 (36.2)	17 (25.4)	50 (26.6)
Gastric or duodenal ulcer	2 (0.9)	3 (4.5)	5 (2.7)
Others	4 (1.8)	3 (4.5)	5 (2.7)
Inadequate indications	170 (42.5)	33 (33)	112 (37.3)
Gastroesophageal reflux	39 (23.1)	8 (24.2)	39 (35.1)
Dyspepsia in >55 years	36 (21.3)	8 (24.2)	21 (18.9)
Abdominal pain	22 (13)	4 (12.1)	13 (11.7)
Others	73 (42.6)	13 (39.5)	39 (35.1)

*p* value: 0.060 (Phase I vs. PCCs with TS), 0.314 (Phase I vs. PCCs without TS), 0.457 (Phase II: PCCs with TS vs. PCCs without TS). TS: training sessions, N (%), PCCs: primary care centres.

**Table 2:** Indications for eradication according to the III Spanish Consensus Conference on *Helicobacter pylori* infection [19].

Peptic Ulcer
Non-investigated dyspepsia in patients <55 years old and without alarm symptoms * (Test and Treat strategy)
Functional dyspepsia
History of peptic ulcer and long-term treatment with NSAID or aspirin
Low-grade gastric MALT lymphoma
Gastric cancer
First-degree family history of gastric cancer
Atrophic gastritis or intestinal metaplasia
Iron deficiency anaemia of uncertain aetiology
Idiopathic thrombocytopenic purpura
Vitamin B deficiency of uncertain aetiology
Offer treatment to all patients with confirmed *H. pylori* infection

MALT: mucosa-associated lymphoid tissue, NSAID: non-steroidal anti-inflammatory drugs. * Alarm symptoms: unexplained weight loss, anaemia, bleeding, dysphagia, persistent vomiting, palpable abdominal mass. Adapted from: Gisbert, J.P.; Calvet, X.; Bermejo, F.; Boixeda, D.; Bory, F.; Bujanda, L.; Castro-Fernandez, M.; Dominguez-Munoz, E.; Elizalde, J.I.; Forne, M.; et al. III Spanish Consensus Conference on *Helicobacter pylori* infection. *Gastroenterol. Hepatol.* **2013**, *36*, 340–374. https://doi.org/10.1016/j.gastrohep.2013.01.011.

## Data Availability

The data presented in this study are available upon request to the corresponding author.

## References

[1] Hooi J.K.Y., Lai W.Y., Ng W.K., Suen M.M.Y., Underwood F.E., Tanyingoh D., Malfertheiner P., Graham D.Y., Wong V.W.S., Wu J.C.Y. (2017). Global Prevalence of *Helicobacter pylori* Infection: Systematic Review and Meta-Analysis. Gastroenterology.

[2] Eusebi L.H., Zagari R.M., Bazzoli F. (2014). Epidemiology of *Helicobacter pylori* infection. Helicobacter.

[3] Burucoa C., Axon A. (2017). Epidemiology of *Helicobacter pylori* infection. Helicobacter.

[4] Leja M., Grinberga-Derica I., Bilgilier C., Steininger C. (2019). Review: Epidemiology of *Helicobacter pylori* infection. Helicobacter.

[5] De Martel C., Georges D., Bray F., Ferlay J., Clifford G.M. (2020). Global burden of cancer attributable to infections in 2018: A worldwide incidence analysis. Lancet Glob. Health.

[6] Shatila M., Thomas A.S. (2022). Current and Future Perspectives in the Diagnosis and Management of *Helicobacter pylori* Infection. J Clin. Med..

[7] Milivojevic V., Rankovic I., Krstic M.N., Milosavljevic T. (2022). Dyspepsia Challenge in Primary Care Gastroenterology. Dig. Dis..

[8] Gisbert J.P., Molina-Infante J., Amador J., Bermejo F., Bujanda L., Calvet X., Castro-Fernandez M., Cuadrado-Lavin A., Elizalde J.I., Gene E. (2016). IV Spanish Consensus Conference on *Helicobacter pylori* infection treatment. Gastroenterol. Hepatol..

[9] Malfertheiner P., Megraud F., O’Morain C.A., Gisbert J.P., Kuipers E.J., Axon A.T., Bazzoli F., Gasbarrini A., Atherton J., Graham D.Y. (2017). Management of *Helicobacter pylori* infection-the Maastricht V/Florence Consensus Report. Gut.

[10] Bujanda L., Nyssen O.P., Vaira D., Saracino I.M., Fiorini G., Lerang F., Georgopoulos S., Tepes B., Heluwaert F., Gasbarrini A. (2021). Antibiotic Resistance Prevalence and Trends in Patients Infected with *Helicobacter pylori* in the Period 2013–2020: Results of the European Registry on *H. pylori* Management (Hp-EuReg). Antibiotics.

[11] Perez I.A., Martinez-Dominguez S.J., Almajano E.A., Carrera-Lasfuentes P., Lanas A. (2022). Management of *Helicobacter Pylori* Infection and Effectiveness Rates in Daily Clinical Practice in Spain: 2010–2019. Antibiotics.

[12] McNicholl A.G., Amador J., Ricote M., Canones-Garzon P.J., Gene E., Calvet X., Gisbert J.P., Spanish Primary Care Societies SEMFyC, SEMERGEN and SEMG, the Spanish Association of Gastroenterology (2019). Spanish primary care survey on the management of *Helicobacter pylori* infection and dyspepsia: Information, attitudes, and decisions. Helicobacter.

[13] Cano-Contreras A.D., Rascon O., Amieva-Balmori M., Rios-Galvez S., Maza Y.J., Meixueiro-Daza A., Roesch-Dietlen F., Remes-Troche J.M. (2018). Approach, attitudes, and knowledge of general practitioners in relation to *Helicobacter pylori* is inadequate. There is much room for improvement!. Rev. Gastroenterol. Mex..

[14] Lin B.S., Li Y.Y., Qiao C., Liu J., Wang J., Wan M., Lin M.J., Zhang W.L., Ding Y.M., Kong Q.Z. (2022). Implementation of WeChat-based patient-doctor interaction in the management of *Helicobacter pylori* infection: A propensity score matching analysis. J. Dig. Dis..

[15] Wang T., Yang X., Li Y., Li L., Liu J., Ji C., Sun Y., Li Y., Zuo X. (2019). Twice daily short-message-based re-education could improve *Helicobacter pylori* eradication rate in young population: A prospective randomized controlled study. Helicobacter.

[16] Zha J., Li Y.Y., Qu J.Y., Yang X.X., Han Z.X., Zuo X. (2022). Effects of enhanced education for patients with the *Helicobacter pylori* infection: A systematic review and meta-analysis. Helicobacter.

[17] Laredo V., Sostres C., Alfaro E., Arroyo M.T., Lanas A. (2019). Management of *Helicobacter pylori* infection at the primary care level. The implementation of specific counseling improves eradication rates. Helicobacter.

[18] Bennett K., Feely J., Thornton O., Dobson M., O’Morain C.A., O’Connor H.J. (2006). Impact of *Helicobacter pylori* on the management of dyspepsia in primary care. Aliment. Pharmacol. Ther..

[19] Gisbert J.P., Calvet X., Bermejo F., Boixeda D., Bory F., Bujanda L., Castro-Fernandez M., Dominguez-Munoz E., Elizalde J.I., Forne M. (2013). III Spanish Consensus Conference on *Helicobacter pylori* infection. Gastroenterol. Hepatol..

[20] McNicholl A.G., Romero J.A., Calvo X.C., Molina-Infante J., Gisbert J.P. (2021). Diagnóstico y Tratamiento de la Infección por Helicobacter Pylori.

[21] O’Brien M.A., Rogers S., Jamtvedt G., Oxman A.D., Odgaard-Jensen J., Kristoffersen D.T., Forsetlund L., Bainbridge D., Freemantle N., Davis D.A. (2007). Educational outreach visits: Effects on professional practice and health care outcomes. Cochrane Database Syst. Rev..

[22] Giguere A., Legare F., Grimshaw J., Turcotte S., Fiander M., Grudniewicz A., Makosso-Kallyth S., Wolf F.M., Farmer A.P., Gagnon M.P. (2012). Printed educational materials: Effects on professional practice and healthcare outcomes. Cochrane Database Syst. Rev..

[23] Boltin D., Dotan I., Birkenfeld S. (2019). Improvement in the implementation of *Helicobacter pylori* management guidelines among primary care physicians following a targeted educational intervention. Ann. Gastroenterol..

[24] Crankshaw S., Butt J., Gierisch J.M., Barrett N.J., Mervin-Blake S., Oeffinger K., Patierno S., Worthy V., Godbee R., Epplein M. (2020). The Durham Initiative for Stomach Health (DISH): A pilot community-based *Helicobacter pylori* education and screening study. BMC Gastroenterol..

[25] Ahmed S., Salih M., Jafri W., Ali Shah H., Hamid S. (2009). *Helicobacter pylori* infection: Approach of primary care physicians in a developing country. BMC Gastroenterol..

[26] Gene E., Sanchez-Delgado J., Calvet X., Azagra R. (2008). Management of *Helicobacter pylori* infection in primary care in Spain. Gastroenterol. Hepatol..

[27] Boltin D., Kimchi N., Dickman R., Gingold-Belfer R., Niv Y., Birkenfeld S. (2016). Attitudes and practice related to *Helicobacter pylori* infection among primary care physicians. Eur. J. Gastroenterol. Hepatol..

[28] Kim B.G., Kim J.W., Jeong J.B., Jung Y.J., Lee K.L., Park Y.S., Hwang J.H., Kim J.U., Kim N.Y., Lee D.H. (2006). Discrepancies between primary physician practice and treatment guidelines for *Helicobacter pylori* infection in Korea. World J. Gastroenterol..

[29] Tshibangu-Kabamba E., Yamaoka Y. (2021). *Helicobacter pylori* infection and antibiotic resistance—From biology to clinical implications. Nat. Rev. Gastroenterol. Hepatol..

[30] Tacconelli E., Carrara E., Savoldi A., Harbarth S., Mendelson M., Monnet D.L., Pulcini C., Kahlmeter G., Kluytmans J., Carmeli Y. (2018). Discovery, research, and development of new antibiotics: The WHO priority list of antibiotic-resistant bacteria and tuberculosis. Lancet Infect. Dis..

